# “I was able to eat what I am supposed to eat”-- patient reflections on a medically-tailored meal intervention: a qualitative analysis

**DOI:** 10.1186/s12902-020-0491-z

**Published:** 2020-01-20

**Authors:** Seth A. Berkowitz, Naysha N. Shahid, Jean Terranova, Barbara Steiner, Melanie P. Ruazol, Roshni Singh, Linda M. Delahanty, Deborah J. Wexler

**Affiliations:** 1 Division of General Internal Medicine, Massachusetts General Hospital, Boston, MA USA; 2 Diabetes Unit, Massachusetts General Hospital, Boston, MA USA; 3 Harvard Medical School, Boston, MA USA; 4 Division of General Medicine and Clinical Epidemiology, Department of Medicine, University of North Carolina School of Medicine, 5034 Old Clinic Bldg, CB 7110, Chapel Hill, NC 27599 USA; 5 Community Servings, INC, Jamaica Plain, MA USA

**Keywords:** Food insecurity, Medically-tailored meals, Type 2 diabetes mellitus, Socioeconomic factors

## Abstract

**Background:**

Medically-tailored meal programs that provide home-delivered medically-appropriate food are an emerging intervention when type 2 diabetes co-occurs with food insecurity (limited or uncertain access to nutritious food owing to cost). We sought to understand the experiences of medically-tailored meal program participants.

**Methods:**

We conducted semi-structured interviews with participants in a randomized trial of medically-tailored meals (NCT02426138) until reaching content saturation. Participants were adults (age > 20 years) with type 2 diabetes in eastern Massachusetts, and the interviews were conducted from April to July 2017. Interviews were transcribed verbatim and coded by two independent reviewers. We determined emergent themes using content analysis.

**Results:**

Twenty individuals were interviewed. Their mean age was 58 (SD: 13) years, 60.0% were women, 20.0% were non-Hispanic black, and 15.0% were Hispanic. Key themes were 1) satisfaction and experience with medically-tailored meals 2) food preferences and cultural appropriateness, 3) diabetes management and awareness, and 4) suggestions for improvement and co-interventions. Within these themes, participants were generally satisfied with medically-tailored meals and emphasized the importance of receiving culturally appropriate food. Participants reported several positive effects of medically-tailored meals, including improved quality of life and ability to manage diabetes, and stress reduction. Participants suggested combining medically-tailored meals with diabetes self-management education or lifestyle interventions.

**Conclusions:**

Individuals with diabetes and food insecurity expressed satisfaction with the medically-tailored meal program, and reported that participation reduced stress and the burden of diabetes management. Suggestions to help ensure the success of medically-tailored meal programs included a strong emphasis on culturally acceptability and accommodating taste preferences for provided foods, and combining medically-tailored meals with diabetes education or lifestyle intervention.

**Trial registration:**

ClinicalTrials.gov NCT02426138.

## Background

Food insecurity, defined as insufficient access to enough food for an active, healthy life [1], is a common problem for individuals with diabetes mellitus [2–5]. Approximately one in five individuals with diabetes report food insecurity, and this prevalence rises among those with hemoglobin A1c > 9.0%, a common threshold indicating poor glycemic control [4]. Food insecurity represents an important barrier to diabetes management as dietary adherence is central to diabetes care [6]. Food insecurity is associated with higher risk for diabetes complications, such as coronary heart disease and chronic kidney disease [7, 8]. Perhaps for these reasons, food insecurity is associated with substantially higher healthcare use and costs in individuals with diabetes [9, 10].

Food insecurity has been viewed as a prominent intervention target in the clinical care of diabetes [11, 12], and evidence supporting strategies that can successfully reduce food insecurity and improve health outcomes continues to grow [11]. Among these promising strategies is medically-tailored meal delivery (MTM): home delivery of fully prepared meals specifically tailored to the medical needs of the individual — including not only diabetes but other comorbidities, such as congestive heart failure or end-stage renal disease [13, 14]. Compared with other food insecurity interventions, such as referrals to community human services resources or provision of a healthy ‘box’ of ingredients at a food pantry, MTM offers additional potential benefits. By delivering fully prepared meals, MTM addresses barriers such as lack of transportation, and lack of time and/or knowledge needed to prepare meals that adhere to complex diets.

Emerging research shows that MTM can lead to improved dietary quality [14, 15], and is associated with improved healthcare use and cost [13]. However, as use of these programs in the healthcare context is new, much remains to be learned regarding how best to design the programs to maximize their clinical benefit.

In this study, we used a qualitative research approach to query participants in a randomized clinical trial regarding their experience with MTM. Our goal was to understand elements of the program that worked well, and elements that could be improved, in order to foster greater clinical benefit and facilitate more widespread implementation.

## Methods

### Data source and study sample

Participants in this study were drawn from the Community Servings: Food as Medicine randomized crossover clinical trial (NCT02426138). The trial has been previously described in detail [15]. In brief, this was a 24-week randomized crossover trial that evaluated the effect of receiving 12 weeks of home delivered medically-tailored meals on dietary quality, conducted from June 2015 to July 2017. Eligible participants were adults (age > 18 years) diagnosed with type 2 diabetes who had a hemoglobin A1c > 8.0% in the last year and reported food insecurity as assessed by the 2-item ‘hunger vital sign’ (*n* = 42) [15]. The ‘hunger vital sign’ is a validated 2-item indicator of food insecurity status, which asks an individual whether “Within the past 12 months [they] worried whether [their] food would run out before [they] got money to buy more” and whether “within the past 12 months the food [they] bought just didn’t last and [they] didn’t have money to get more.” [16, 17] Meals were prepared from scratch at Community Servings (https://www.servings.org/), a not-for-profit, community-based, medically-tailored meal delivery organization. Frozen and/or refrigerated fully-prepared meals were delivered weekly by delivery truck, with their composition determined by a registered dietician nutritionist to be appropriate for the medical needs of the individual. Ten meals, representing approximately half of the weekly food intake of the participant, were provided each week as single delivery. An example of a day’s worth of meals is provided as Additional file 1: Table S1. Because this trial was designed to test the effect of meal receipt itself on dietary quality, nutritional education was not provided, though it is a component of typical MTM programs outside of clinical trials. After completing the 12 weeks of medically-tailored meals (MTM) provided in the trial, individuals were invited to participate in a qualitative telephone interview to discuss their experience with receiving MTM. Interviews were conducted from April to July 2017.

The study protocol was approved by the Human Research Committee at Partners HealthCare. Written informed consent was obtained from all participants for trial participation, and verbal consent for participation in the qualitative telephone interview was obtained separately.

### Availability of data and materials

Because this study relies on transcripts of oral interviews reflecting personal experiences, and thus cannot be de-identifed without fundamentally altering the data itself, data cannot be made publicly available. Data are available from the authors on reasonable request and with the agreement of the study institutional review board if assurances of protection of participant confidentiality can be made.

### Qualitative data collection and analysis

Our goal was to conduct structured in-depth interviews with a sufficient number of trial participants to receive adequate feedback regarding their experience with an MTM intervention. We first developed an interview guide (Additional file 1: Table S2) using an iterative process that incorporated the perspectives of study team members and participants, and drew from past work in this patient population [18]. The interview guide was designed to assess participants’ experience and satisfaction with the program and to identify areas of improvement. We then conducted semi-structured telephone interviews with study participants, using purposeful selection to enhance age, gender, and racial/ethnic diversity of those interviewed. We continued contacting participants until content saturation was reached. We anticipated content saturation would be reached after interviewing approximately 1/3 to 1/2 of intervention participants [19]. As described below, coding of interviews occurred during data collection, to determine whether new codes were being added. As we approached 20 interviews, the number of new codes added per interview decreased substantially, leading us to conclude content saturation had been reached. The interviews queried the participants about the meals received and their perceptions of the influence of the meals on diabetes management. We sought suggestions regarding how the meals could be improved and alternative resources that may assist with diabetes management for individuals with diabetes and food insecurity. Interviews lasted approximately 20 min (range: 15 to 40 min). The interviewer (NS) used reflective probes to motivate participants to elaborate on their comments. Interviews were audio-recorded with permission from participants, and recordings were transcribed verbatim. We provided participants with a $20 store gift card for their participation.

### Quantitative data

In addition to collecting qualitative data, we extracted sociodemographic and clinical information from the trial baseline examination and questionnaire to better describe the study sample. Details of these assessment procedure have been previously described [15]. We measured height, weight, and blood pressure using calibrated instruments and a standardized measurement technique; we also performed phlebotomy for laboratory assessment of hemoglobin A1c and serum lipid levels.

### Data Analysis

Transcripts were coded and analyzed using the content analysis approach [20]. We used an integrated inductive-deductive technique to construct our coding framework [21]. The interview guide and an initial review of the data were used to create a preliminary list of codes and sub-codes. This approach allowed us to produce an organized framework, while giving us the flexibility to include new codes as they emerged. Each transcript was coded, using NVivo 11 software (QSR International, Melbourne, AU), independently by two investigators: the interviewer and the principal investigator of the study. The two coders met weekly to ensure consistency and transparency in the coding. Discrepancies were resolved through reflection and dialogue until consensus was reached. Relevant quotations were selected to illustrate key points within each theme.

## Results

### Participant characteristics

We reached content saturation after interviewing twenty individuals from the Community Servings: Food as Medicine randomized crossover clinical trial (NCT02426138). This represented approximately half of the total number of trial participants. The mean age of the included individuals was 58 (SD: 13) years, and 60.0% were women (Table 1). Twenty percent of the participants were non-Hispanic black, and 15.0% were Hispanic. The mean of the participants’ income, expressed as a percentage of the federal poverty guideline for their year of study enrollment and household size, was 147% (SD: 54%; as an example, for a single individual in 2016, the federal poverty guideline income threshold was $11,880). The mean duration of diabetes among participants was 13 (SD: 10) years, the mean baseline hemoglobin A1c was 8.0% (SD: 1.6%) and mean body mass index was 34.7 kg/m^2^ (SD: 6.2 kg/m^2^).

**Table 1:** Demographic Characteristics of Participants

	*N* = 20
Age, years, mean (sd))	57.6 (12.6)
Female, N (%)	12 (60.0)
Race/Ethnicity, N (%)	
Non-Hispanic white	13 (65.0)
Non-Hispanic black	4 (20.0)
Hispanic	3 (15.0)
Education, N (%)	
< HS Diploma	1 (5.0)
HS diploma	4 (20.0)
> HS diploma	15 (75.0)
Income as Percent of Federal Poverty, mean (sd))	147 (54)
Insurance, N (%)	
Private	3 (15.0)
Medicaid	5 (25.0)
Dual	12 (60.0)
Born outside US, N (%)	8 (40.0)
Duration of Diabetes, years, mean (sd))	12.9 (9.6)
Hypoglycemia in last 3 mo^a^, N (%)	8 (40.0)
Reports cost related medication underuse^b^, N (%)	5 (25.0)
Reports making food and diabetes medication/supplies tradeoffs^c^, N (%)	6 (30.0)
SNAP participation in last 12 mo^d^, N (%)	14 (70.0)
Self-reported health status ‘excellent or very good’^e^, N (%)	12 (60.0)
Baseline Hemoglobin A1c, %, mean (sd))	8.0 (1.6)
Baseline Low Density Lipoprotein Cholesterol, mg/dL, mean (sd))	109.6 (42.9)
Baseline Total Cholesterol, mg/dL, mean (sd))	186.3 (46.8)
Baseline High Density Lipoprotein Cholesterol, mg/dL, mean (sd))	45.3 (12.4)
Baseline Triglycerides, mg/dL, mean (sd))	157.4 (84.1)
Baseline Systolic Blood Pressure, mm Hg, mean (sd))	134.4 (23.6)
Baseline Diastolic Blood Pressure, mm Hg, mean (sd))	80.3 (10.3)
Baseline Body Mass Index, kg/m^2^, mean (sd))	34.7 (6.2)

^a^Indicates self-report of symptomatic hypoglycemia, with or without requiring assistance from others; ^b^indicates self-report of not taking medications as prescribed, taking less medication, or not filling medications owing to cost; ^c^indicates self-report of putting off buying food in order to buy diabetes medications or supplies, or vice versa; ^d^SNAP = supplemental nutrition assistance program; ^e^response options are ‘excellent’, ‘very good’, ‘good’, ‘fair’, or ‘poor’

### Satisfaction and experience with MTM

Overall, participants reported being satisfied with the MTM program (illustrative quotes are in the main text with additional quotes in Table 2). One participant said “*I was glad that I was able to participate, and that the drivers provided the ease with the meals, and it’s exciting to get meals weekly. It kind of alleviated the headache of either buying or cooking. You know? And when you knew it was a delivery day, you had peace of mind for the rest of the week [laughter].”* Participants noted positive interactions with the study staff. Regarding meal delivery, many participants were pleased with the convenience of home-delivered, medically-tailored meals, though a small number expressed difficulty coordinating meal deliveries due to their work schedule. Of note, this program did not require participants to be homebound. Most participants were satisfied with the amount of food they received over the course of the study, stating the food typically lasted for 5 days, the intended amount of time. Regarding the amount of food for a given meal, most participants found the portion size adequate and reported feeling full after finishing a meal.

**Table 2:** Themes and Illustrative quotes

Themes	Quotes
Satisfaction and experience with MTM	
Convenience	The most, I like the special diet I was on for 12 weeks. It helped me a lot and it was done in the very easy for the customer manner because the food was delivered to my door. So this is what I like the most. What did I like? It was very convenient because I don’t get out too much. And I have no way-- there’s a shuttle bus that comes here once a week that can take you shopping. But sometimes if the weather’s bad or if I don’t feel good, I don’t even get to go. And I live alone so I found it very convenient that they came to the door with those meals. Sometimes I don’t know what I would’ve done without them. Pretty convenient, it did-- I have to say it did cut into what I can do on Tuesday, which was the day that sometimes I do have other things. So, again, I would have preferred Friday which was a much easier day for me to do deliveries. But I know, again, that they only had a certain day that they could do it. So I’d say that was the one thing that was a little bit difficult for me, for the whole participation.
Interactions with Staff	The Community Servings people were fantastic. The only problem I had was the first or second delivery. There was no smaller time frame, it was from 8 AM to 4 PM or whatever, the first couple of times. But once they got figured out where I was on a schedule, then it was very easy. Very accommodating. Yeah. They were very friendly. When I asked about the whole switch of the egg option from the menu, we weren’t able to change it because it was already in place, but she tried. So that was nice of her.
Food Quantity and Proportion	I liked the fact that they were prompt and liked the fact that there was a quantity of food, enough food to kind of-- almost food for a week. Oh, yes. And as I said, each meal was satisfying, fulfilling. It was not too much, not too little. When I was finished the plate, I was not left wanting.
Food preferences & Cultural Appropriateness	
Quality of Meals	Yeah. It had spice in it. It had flavoring. It wasn’t flat. See, we will get meal from wheels from the city, but they don’t look out for our health. They give us things that are loaded with sodium, with potassium, carbohydrates. They don’t watch your diet. So this package that you gave to me helped me out immensely. A number of things. First of all, I liked the variety of foods and the quality of the foods. It gave me some good ideas about things that I could prepare for myself. That there was a choice of variety in the food. And that it came with the meal, with the dinners, and you also got soups and snacks with it. I’m glad that there was a lot. You go into a meal that-- you had the balance, I guess. Like I said before, the rice thing; get rid of that. Well, for at me at least. Oh, so I just remembered this. A few times they give you the same meals in the same week. They might give you, we’ll say, like two turkey meals or something, whereas the other ones, they would give you a different one each for five days. I think that if they had a normal diet, it would really be helpful for diabetes. And it would be helpful, that especially me to have a meal at night, you know to be able to have a meal at night and just heat it up. But I think it has to be something that is normal. You know, a normal type of meal. I don’t know what they put in-- I don’t know what they put in the meal.
Nutritional Value	They help me stay away from what my nurse from other hospital said I shouldn’t use for diabetes, like white food. For example, white potatoes, white rice, white pasta; they stay away from this. They gave me vegetables, a lot of salads. It was done for diabetes. They were bland and what I needed to have at that time. I would not have done such a good job at home with what I needed to get healthy, whereas they were on target, did the right thing, where I probably wouldn’t have done that at home. So, for six weeks, it really got me in a great place and healthy, and it made all the good choices for me. It may have been better because I’m diabetes so what they bring me help me with my diabetes. Because I buy it. Like I said, I bought them. I cooked them myself, and they helping me. Like everything I see them cook, and then I bought it now, and they helping my blood pressure go down a little bit. Yes, I think that they could have dedicated it more to a diabetic. I mean, I think most diabetics, eat a piece of meat, and a vegetable, or a potato, or a salad. And this was just food that I never ate before.
Cultural Tailoring	For me it’s like spice. Put more spice on it. I feel like it just doesn’t have enough spice on it to taste good. Yeah, I think that they could have been chicken, and fish, and meatballs, or a lamb chop, or something normal.
Diabetes Management and Awareness	
Access to Healthy Meals	It’s actually helped me out a lot because they cut me down on food stamps, so I was actually unable at the time to be able to eat the foods I was supposed to. So having those meals was perfect timing and I was able to eat what I was supposed to eat. And you can open up my refrigerator and there’s food there. Not that there wasn’t food before, but when I went out, I’d spend a lot of money on junk and foods that maybe I shouldn’t have been eating. But it did, it helped me out tremendously, financially. It really, really did. Perhaps what I think of as a happy meal, but if we buy our own food, we tend to shop the discount centers in the stores, the day-old bread that’s cheaper. We basically live off the discount corners in the stores or the food pantry and the senior lunches that we get. I guess what I’m trying to say is, lack of funds often reduce the choices in our meals. I think that helped me in many ways because some people have diabetes-- some of them they can’t afford that the nutrition they want them to eat. When Community Servings have them send them the food, they help them out, to help them, you know, in a way you can manage your diabetes.
Money Management	Yes, it did. It just saved me not being broke for the rest of the month to pay my bills, actually. Oh, you don’t know, honey, you don’t know. I was down. I cut out a lot. I cut out the channels on the television, the computer because we’re living on limited income, and they take a third of it for our rent, a third of it for if you have half the package with the Internet and everything. So it did help me. At the end of the month, I could put my hand in my pocket and have $10. And that’s a good feeling. Yes, they did. My particular case, yeah, they helped me save money. It did actually really help me save money.
Health Promotion	I would say, just, it was a helpful reminder about eating right, and taking care of myself, and taking medication properly, and all of that as part of a comprehensive way of living, so eating and doing things that are helpful for the diabetes, which is a way of sort of helping promote that. My diet. To control myself because before, I knew I’m diabetic, but sometimes I didn’t have a choice, in what I ate in the day. But soon I started this program, I take it serious. Well, another thing I was gonna tell somebody was that-- was keeping a record, that’s was really a good saying. It’s hard to keeping a record of what eat, when you eat was really a good idea. Because you have a little bit more time. But I think manuals of keeping your record manual, they’re really important. They’re really great. You have to educate yourself to it. Take a look at what you’ve done to have accountability because you-- if you live in a society with a lot of fast food and a lot of convenient food had been and I think, your record has been very good. Your record would have to spin to mind to a certain way of thinking. And your records were really good. I liked your records. A little bit hard to fill out, but I liked them. It’s a little bit time-consuming, but I like the idea of having records.
Improvements in Health	Well, this diet definitely brings down my sugars. When I started the program the first time-- the program that is-- which I think this interview may be part of, it brought down my sugars from an A1C of 10-something to 9, in the 3 months that I was on it. My AC1 or A1C, whatever it’s called, dropped significantly. I was over the seven something, and now I’m under. I’m in the six something, six point something-- I don’t know the numbers off the top of my head, I’m sure you have it in the records. I used to give myself four shots of insulin a day, and now I just take a pill in the morning. So that is a huge difference in my life. I noticed when I tested my blood, which I did on a regular basis, my numbers were lower than before. It took a few weeks to see the difference, of course. But I noticed that. And I didn’t feel as fatigued, in a way. I had a little more energy, also. Well, I guess it helped me understand that healthy food makes me feel good, health-wise. Whereas, let’s say on a Wednesday night when I have Bible study, I would have to eat out. I would notice the difference.
Weight Management	It does help to manage the weight, also because I’m getting pre-portioned package of food, A. B, I actually get more food than I would have eaten. And so I relied more on carbohydrates in the past, that are cheap, affordable, and easy to make and eat. Whereas, this is definitely an asset. Well, everything is portioned so it did make it easier. And again, the whole variety of if I don’t want to do a dinner for work, I’d do the stew and then the apple for snack. So it helps manage my weight. Whereas, if I bought something from the cafeteria at work, then you know how everything is oversized. It helps keep everything in perspective and in good portions, which helps with the weight. Oh, it certainly managed my weight and helped me lose a significant amount of weight, because, it was all-- everything was determined for me. And I know they were determined based on what my need was, and, because of that, those-- I didn’t have make those decisions, and there was a packet with all the meals, explaining what’s in it and what’s on-- so that was a significant help.
Model for Food Preparation	Well, usually the couple of meals each that I liked a lot, and I wrote down-- I kept a diary and I wrote down what my favorites were for each. They had some interesting ideas, like using barley or bulgur wheat which I hadn’t really thought of, and the portions were also helpful to see what was recommended for somebody with diabetes. So it wasn’t just the quality of the meals, or what was being served, but also the size of the meals was helpful to me, to see what was allowable and also the various kinds of things. It was helpful to see what was acceptable for somebody with diabetes. Well, it’s as I said, following the example of both their ingredients and their preparations was a very useful educational tool, kind of like setting the example of what we should be eating. And learning proportions, which is a biggie, following the example of the size of the proportions helps also when eating on our own, not over-filling the plate. But it made me actually see what ideas I can incorporate to help me measure stuff out, because sometimes I do tend to overeat, and that helps me get an idea of what to use as a mental measurement when I’m proportioning meals out from whatever we’re fixing for supper. And I hardly eat that stuff anymore. That’s Italian stuff that I was brought up with, but I noticed lately it bothers my stomach. I stopped eating all that and I eat more salads. Like I’ll eat in the morning-- like yesterday I had eggs, but most of the time I like cereal. I like oatmeal. And I love that quinoa, and that’s something new I’m eating. I learned what to prepare. I don’t cook a lot of rice. I see when they bring the food, I see what kind of thing they put is good for diabetes. I put it myself. Now I cook it for myself. Well, the portion size. Because when you cook for one person, it’s harder to-- because I was used to feeding people [laughter]. But cutting down on the portion size. And I didn’t realize doing that doing that, you can still feel satisfied. You don’t have to have a whole bunch, like a cup of this, where you can have half of it. So that kind of taught me I was satisfied with the meals and everything, and I didn’t need all that much food to feel full. I think it’s helped me think about options that are healthy and, particularly, buying fresh foods and cooking it myself, and also, the mix of foods, the protein, the starch, and the vegetables. I think just eating that over the course of a number of months, you just get used to doing that. And so, you sort of become more used to that. And also, fresh vegetables and fruits, I’m thinking more about eating those. So, yeah, I think it was a good way to sort of get me on track towards eating them. They taught me how to eat because we’re not told enough about diabetes. I was just told I have it, and they send me to a nutritionist, but like I say, all they do is give you a printout list of foods to pick from. It wasn’t chosen for you. So, you can only hope to God you’re eating the right things. I’ve learned to spice chicken up now, And I’ve learned different things that they did that is still healthy instead of just plain old chicken, thinking that was what I had to do, and it was frustrating, and it was boring, and it made me want to cheat, whereas now I’ve learned, because of the prepared meals, how to try different things. So I think that’s a positive influence. Yeah, because I can see what I have before. And when I have the food to see how I can prepare my food better, that is a good experience.
Difficulties to Diabetes Management	Mostly, that I can’t-- I’m physically unable to exercise, and that’s the second part of fighting, combating diabetes. So I would say it has been very little to do with the program, and more to do with my physical limitations. I still have a little bit of trouble preparing a wide range of different foods, and the foods that the Community Servings didn’t cover. I still have to make my own foods. And although it has been a help, especially it’s also been a financial help. I’ve had to spend less money purchasing food, so that was certainly a consideration. So I would say that those are the things that I would mention.
Suggestions for Improvement	
Seasonings & Spices	Yes, some people like spicy foods, some people don’t. Like when you making the fish, the island people like the fish spicy. Or maybe you can make it have some barbecue chicken. That would be good, too. They have red hot pepper. You make a little bit and you give those food a little more flavor. I like garlic. Cocoa, garlic, and parsley, stuff like that. I make my own spice. I put green pepper and white pepper all together and-- but they didn’t have all that on their foods. Well, right now I’m having a water retention problem. My legs and my feet are swollen. So I’m really trying to lay off the salt. I don’t know how much sodium was in those meals, but I would say less salt.
Food options	The only thing I’d have to say is more of a selection. It was a good selection, but it seemed like every now and then it would be the same thing. The breakfast options would be important for certain lifestyles that people have. I think it’s an important lifestyle thing. The breakfast every morning is so important. Okay? I’ve already stated that I do think that they should be correlated more to the weather. They do occasionally have a chicken salad or a tuna salad, maybe they would do more of those in the summer months when you really can’t eat a heavy beef meal or-- fish is always, of course, appropriate in the summer. But some of the heavier chicken meals or beef meals seem inappropriate for a day that’s humid, where your appetite is just diminished. And I would like to see - but I know this is food that is in part contributed to Community Servings, so it’s a little hard to be demanding - but it would be nice if there were more fresh fruits. There’s an occasional apple and an orange, but it would be nice if there could be more fresh fruits. I’m a fruit lover so I would’ve liked more fruit. And they only gave you two yogurts, and I love yogurt. Sometimes I eat that for lunch as a meal. Yeah, I would’ve liked more yogurt. I told you just maybe explain those meals on the container or receipt when they make something foreign, like some ethnic food from another country or something like that because I didn’t know what I was eating. No, just find out if the person has an allergy before they sign up for the-- I mean, when they going to get into the program. Then if they have a head start that way, it helps.
Meal Quantity	But what I’m saying is that can be improved. The quality is good, but the amount of the food, it could be a little bit more. If they want to really want to have a good-- how can I say? They really make an improvement to the patient, you know? I did think those cereals, by the way, were a bit small. I thought the cereals were tiny. The cereals just only last-- cereals were very small. They lasted maybe one time or something. So, yeah, so asked for more foods. And the desserts were-- they gave you that sliced pound cake sometimes with some peaches or something like that. But it was only a scanty little, little thin slice. But I know they’re diabetic meals and they’re counting calories and all that. But I would’ve liked a bigger piece of pound cake.
Suggestions for Future Assistance	
Diabetes Education and Support Groups	I would take anything they gave to me, to be honest with you, really, because like I say, I learned a lot about diabetes, and I didn’t have that before. I’ve seen people in my building that had diabetes that have lost their legs from the way they eat, the way they don’t exercise. So, I mean, you’re learning more about the disease itself, which we’re not told at all. And it’s we’re just getting my Metformin, and that would be it. I think that would be enormously because it’s very clandestine. If I don’t see it-- if I didn’t see it in other people, and see if it’s effectual to people, I probably would be less-- a little bit more careless. I try not to be careless. I’m not going to try to postpone it anymore. I’ve seen the effect of diabetes in other people. I see people losing their legs, so I’m aware of that. Community support groups are important I think. Probably a support group with other people that are in the same boat. Well, I have read that diabetes is caused by a slight infection in the pancreas. I think addressing that issue would have a long-term effect on diabetes patients in general.
Financial Assistance with Medication and Food	Yes, that will be good because some medication very expensive, some patient they can’t afford it, but if they can-- any way they can help them with the medication, that would be wonderful, too. Well, I have all of that available to me through my PCP. Except when you say medication assistance, is that financial assistance? I most probably could use help with that. I do only have my Social Security, is what I’m living off of. And I think most people know that’s very hard to live off of, but I guess that would be what I would be interested in. That would be something I’d be interested in, because those things are very expensive. Yeah. Yes. So the meals in hand with the medication management would be super helpful. I think it would be great if there was a special kind of program that could disperse some money to help diabetics get the food that they need. Because the food stamps, people don’t really care-- not getting into that, but they go by your income. And even though your income is so much, they don’t realize the bills that you have pay. So if there was some kind of grants, or something, or some special store just for diabetic, that would be great. The only thing I would like to add is, is there a way that they can give people the food, and they can make it themselves? That’s the only thing I would like them to do. Not cook it, and just bring it to them that they can make it themselves? I would say the nutrition counseling. Fitness would be another one. And the other thing that could help me a lot would be trying to get food, you know? Because it’s one of my major problems is getting the right food.
Fitness and Nutrition Programs	Yeah. The fitness program because I think it all comes together in a way. It’s like stop eating as much and reduce the food. Yeah, you can do that. But I think exercising also increases muscle. Because you start losing muscle and stuff like that and with the exercise, you’ll be to keep your-- going and plus even lose faster than just reducing calories and stuff like that, too. Nutrition counseling would be good. I would love to go to-- what do you call someone who just deals with vitamins? I am a very strong and firm believer of vitamins and I take a lot of them, and the doctor says that’s what keeps me going. I think nutritious counseling would be excellent. I always think the program--- although you mentioned something about physical therapy. I think that would be magnificent. I’m the type of person where growing up, I was an exercise freak. After the bad, bad accident that made me disabled, I tend to not to do exercise routines on my own unless I go to the gym or I go to the physical therapist. For me, that would be a big one. The nutrition is good, and the people can talk to tell you how much you can do the exercise, the portion of food you can eat, what kind of food you can eat, what kind of thing you can do for helping you with the diabetes.

### Food preferences and cultural appropriateness

Most participants enjoyed receiving medically-tailored meals. Many of the participants were satisfied with the quality of the food offered during the intervention, often describing their meals as “healthy” and “balanced”. One said, “*I thought they were very well prepared. They were tasty and they were healthy, obviously. And it just helped to be able to get a full healthy meal all in one, with one thing. Like, “Here’s everything you need for one healthy meal [laughter].”* Participants also reported being pleased with the variety of food options that were provided throughout the intervention.

Participants emphasized certain aspects of the meals that promoted acceptability. In particular, participants favored familiarity, selection, and healthfulness of the meals. Most participants recognized that the meals provided were appropriate for those with diabetes. However, some did not, which underscores the importance of nutritional education in conjunction with MTM. One participant said, *“Some of the meals, I didn’t even know what they were. Even though it gave you the name, I didn’t know really what it was. I think it was some of the grain meals, and it was a meat and it was lamb or something. There were a few things I didn’t like because I don’t eat.”*

Given the diversity of the study sample, we explored whether the meals met the cultural needs of the participants. Most participants reported that meals were acceptable, but some expressed interest in receiving meals representative of their country of origin or cultural background. Overall, participants emphasized the importance of having food options that reflected their culture and thus felt familiar and appropriate, with one noting *“As I said I am from Haiti. Sometimes some food they gave me, I eat them, they’re good for my body. But I think maybe if they can maybe add a little bit more island food, that would be much better.”* Participants reported that culturally acceptable meals may facilitate sustaining adherence to a healthy diet after MTM participation ends, as this would make it easier to incorporate healthier food into their usual diet.

### Diabetes management and awareness

Participants reflected on how the medically-tailored meals allowed them access to nutritious meals they otherwise would not have been able to afford. One summarized, *“I understand that it does better on this diet. But these are foods that I would not be able to afford to make for myself, or be physically capable of making for myself. The question’s a little more complicated than that. I mean, I understand diabetes and I understand what foods work and what don’t work. But being that I have a spinal cord injury and can’t really cook a lot for myself, and shop a lot for myself, and don’t have an income, I guess the answer is yes* [the question being asked is if the program helps the individual adhere to a healthy diet]*, but it’s not like I don’t know what foods are appropriate. I can see these foods and the foods that I’m eating are appropriate for fighting diabetes and keeping it under control. But I wasn’t unaware of that, I just needed someone to be able to help me to do this.”*

Furthermore, they described an element of ‘modeling’ such that having experience with diabetes-appropriate food helped them to understand appropriate portions and food components in a healthy meal. One participant noted *“Well, I really like how much food they give it to me and they teach me how much I have to eat every single time I have to eat,”* while another said, *“I liked the portion control because there was everything that you needed on that plate, and it wasn’t a big portion but it did make you full. And that’s what I liked best about it. And it taught me how to go out and buy certain things that were on the plate so that I could eat separately.”*

Participants commented on multiple sources of expense in their lives, including both diabetes-specific expenses such as medication and supplies, along with basic necessities such as housing. Participants reported that participation in the medically-tailored meal program was helpful for overcoming these barriers, since they did not have to allot as much of their own resources to food. This meant that they could better budget for other elements of diabetes management, such as affording medications, or simply other household necessities. *“Oh, you don’t know, honey, you don’t know. I was down. I cut out a lot. I cut out the channels on the television, the computer because we’re living on limited income, and they take a third of it for our rent, a third of it for if you have half the package with the Internet and everything. So it did help me. At the end of the month, I could put my hand in my pocket and have $10. And that’s a good feeling.”* Participants also reported that the support of the program reinforced their efforts in diabetes self-management, and may have augmented self-efficacy.

Participants noted several benefits they perceived from program participation, aside from diabetes management itself. Participants were relieved of the stress and burden of following a complicated diet. Also, since they no longer had to worry about obtaining nutritious food, participants reported being better able to manage other responsibilities in their lives, and to allocate their attention to managing diabetes.

Regarding diabetes management, participants reported improvements in weight, and in biomarkers of diabetes control, such as hemoglobin A1c, with one saying, *“So what I’ll tell you was when I started my [A1C] level was so high. And when I started this program they had to teach me how much food I have to eat and all the protein I have to get. And then through the program my A1C went down. And my PCP, they tell me everything that was perfect and that she’s so happy, and I’m so happy right now I know how I have to eat.”* Despite these benefits, some participants continued to struggle with other obstacles that made it difficult to manage their diabetes, including physical disability, medication regimens, and financial pressures not alleviated by MTM. Attempts to address these additional barriers will likely be important components of future interventions.

### Suggestions for improvement and co-interventions

Participants had several suggestions regarding how to make MTM programs effective, and how to combine MTM with other interventions to help manage their diabetes. Participants strongly emphasized the importance of having food that reflects their cultural background and preferences regarding seasoning and palatability. Beyond meal provision, individuals believed combining MTM interventions with diabetes self-management education or lifestyle intervention could have a synergistic effect on improving diabetes management. Diabetes support groups and adding physical activity and fitness components were also suggestions for future interventions. Finally, assistance with other financial barriers to diabetes management beyond food – in particular medications – was noted as an important area for future programs.

## Discussion

Semi-structured interviews of medically-tailored meal delivery program participants revealed that participants perceived several positive effects, including improved quality of life, ability to manage diabetes, and stress reduction. Participants also had suggestions of improvement for future interventions. Participants suggested combining MTM and diabetes self-management education, or a lifestyle intervention, and providing additional financial assistance, particularly with medications. Regarding the meals in particular, participants emphasized the importance of receiving food that was culturally appropriate with acceptable taste and familiar ingredients.

This study is consistent with and expands our knowledge of medically-tailored meal delivery programs and corresponding health outcomes. Prior studies have suggested that meal delivery programs can improve dietary quality [14, 15], reduce aspects of distress related to living with illness [14], and improve symptoms [22], largely based on quantitative assessments of these features. This study adds a qualitative understanding of the results of MTM participation. In addition, it offers direction for those looking to enhance MTM programs by making them more effective for achieving clinical outcomes.

This study has several implications for future research. Critically, it highlights the opportunity for joint intervention, particularly in combining MTM with education and/or lifestyle interventions. These types of joint interventions may offer a more comprehensive package to those individuals living with diabetes and food insecurity. The skills learned in the lifestyle component of the intervention could potentially enhance the sustainability of intervention effects after conclusion of meal delivery. The study also highlights substantial financial barriers those with food insecurity face, even apart from access to food. Addressing the multitude of health-related social needs faced by these individuals offers additional avenues to improve health and diabetes outcomes. Two key areas for investigation that were not discussed by participants include social isolation and the duration of intervention. With regard to social isolation, prior work on non-tailored home meal delivery, which typically includes daily meal delivery with a home visiting component, has shown reduced loneliness as a benefit of the intervention [23, 24]. For MTM programs, which typically have less frequent (e.g., once weekly) delivery and may not have an explicit home visiting component, it is unclear if these same effects occur. The appropriate duration of intervention for MTM is also unclear. Prior work from our group has shown that, when limits are not imposed, participation occurs for approximately 12 months [25]. Whether shorter or longer durations would be beneficial remains to be determined. Finally, responses from this study highlight the need to examine patient-reported outcomes in MTM program evaluation. Focusing solely on clinical biomarkers or healthcare utilization likely overlooks important benefits perceived by the participants. Whether current instruments that assess patient-reported outcomes are adequate to this task, or instruments specific to the circumstances of the patient populations that receive MTM interventions are required, is an important question for future study.

The results of this study should be interpreted in light of several limitations. This study only analyzed a portion of participants in one MTM program, from a circumscribed geographic area. Though sufficient to reach content saturation and diverse in racial/ethnic background, these results nevertheless may not generalize to other settings. Additionally, while the experience with the intervention reported in the interviews was strongly positive, with concerns raised primarily about food preferences, those who had more negative experiences may not have wanted to be interviewed. However, we regard these findings as hypothesis generating for future studies. A key factor that this study did not fully address was the effect of MTM on households, as opposed to individuals. In this study, the majority of participants lived alone, but because food insecurity is experienced at the household level, the experience of other household members is certainly relevant. Effects on the household may be especially relevant when members are caring for young children. These limitations were balanced by key strengths, including in-depth qualitative examination of a participants in a novel intervention program, and direct feedback from participants to improve future MTM.

Though further research, particularly longer and larger-scale randomized trials, is needed to demonstrate the effectiveness of MTM interventions for health outcomes, it is also important to consider how widespread implementation may be achieved if effectiveness is established. Recent innovations in healthcare financing provide a pathway to sustainability as a covered health insurance benefit [26]. Medicaid demonstration projects in several U.S. states are incorporating MTM into their programs [26–28], and recent changes to the Medicare Advantage program also offers opportunities to make MTM more available to beneficiaries [29].

## Conclusions

Medically-tailored meal delivery programs are a promising approach to managing a difficult clinical problem—how to improve health in those with both diabetes and food insecurity. Medically-tailored meal programs offer the potential to improve not only biomarkers, but also patient-reported outcomes that are important components of quality of life. Themes identified in this qualitative evaluation can be incorporated in future interventions to help improve health in vulnerable individuals with diabetes.

## Supplementary information

**Additional file 1: Table S1.** Example of 1 day of medically-tailored meals. **Table S2.** Telephone interview guide.

## Data Availability

Because this study relies on transcripts of oral interviews reflecting personal experiences, and thus cannot be de-identifed without fundamentally altering the data itself, data cannot be made publically available. Data is available from the authors on reasonable request and with the agreement of the study institutional review board if assurances of protection of participant confidentiality can be made.

## Abbreviation

MTM: Medically-tailored meal
