# Supplementary material for: “I was able to eat what I am supposed to eat”-- patient reflections on a medically-tailored meal intervention: a qualitative analysis
Source: BMC Endocr Disord. 2020 Jan 20;20:10. doi: 10.1186/s12902-020-0491-z (PMC6971854; doi:10.1186/s12902-020-0491-z)
Supplement: Supplementary file 1 — Additional file 1: Table S1. Example of 1 day of medically-tailored meals. Table S2. Telephone interview guide. [file 12902_2020_491_MOESM1_ESM.docx]

Additional file 1

**“I was able to eat what I am supposed to eat”:**

**Patient reflections on a medically-tailored meal intervention**

Seth A. Berkowitz, MD MPH; Naysha N. Shahid, BA; Jean Terranova, JD; Barbara Steiner, EdM; Melanie P. Ruazol, BA; Roshni Singh, BS; Linda M. Delahanty, MS RDN; Deborah J. Wexler, MD MSc

| Table S1. Example of 1 day of medically-tailored meals | |
| --- | --- |
| Breakfast | Hard-boiled eggs |
| Lunch | Asian noodle salad  Lentil Vegetable soup  Apple |
| Dinner | Herb-roasted perch with polenta and asparagus  Fresh fruit (apple, orange, or pear) |
| Snacks | Greek yogurt |

**Table S2.** Telephone interview guide

1. How satisfied were you with the Community Servings (CS) program?
   1. What did you like about community servings?
   2. What didn’t you like about community servings?
2. Tell me about your experience coordinating with the Community Servings team.
   1. *Probes: Were you able to reach them when necessary? How would you describe the employees? How accommodating was CS? Were the informational handouts from CS useful?*
3. How would you rate the service provided by Community Servings?
4. How convenient were the meal deliveries?
5. Tell me about the meals Community Servings provided.
   1. What did you like about the meals?
   2. What didn’t you like about the meals?
6. Was the amount of food delivered each week sufficient?
   1. *Probes: Did it last over a week? Did you ever feel like there was not enough food? Were you full after finishing a meal? Did you snack or eat other foods in addition to the CS meal?*
7. Would you have preferred the meals to cover everything you ate in a week, rather than half your meals?
8. What suggestions can you provide about how to improve the quality of the meals served?
   1. *Probes: Taste, freshness, meal options, types of foods?*
9. Is there any way the meals could have been better tailored to your medical needs? If yes, how?
   1. How about your cultural needs? If yes, how?
   2. *Probes: Are there any specific foods or spices that CS could have been included in the meals that are good for your health? Were the meals nutritious?*
10. Could you tell me about how, if at all, receiving the meals has changed the food you prepare for yourself, or what you think of as a healthy meal?
11. Could you tell me about ways that receiving meals helped you manage your diabetes better?
12. Could you tell me about ways participating in the program may or may not, have helped you understand your diabetes better?
13. What difficulties with managing your diabetes did you still have despite receiving the meals?
    1. *Probe: Did receiving the meals help you save money for other necessities?*
14. Tell me about ways receiving meals did or did not make it easier to manage your weight.
15. In addition to programs like Community Servings that help patients manage diabetes by providing healthy meals, what other resources would you like offered to assist with diabetes management?
    1. *Probes: Case management, fitness programs, medication assistance, nutrition counseling? Would you be interested in enrolling in another program similar to CS in the future?*
